# Lung Ultrasound Findings in Pediatric Mycoplasma Pneumoniae Pneumonia: A Prospective Multicenter Pilot Study

**DOI:** 10.3390/children12121669

**Published:** 2025-12-08

**Authors:** Mariantonietta Francavilla, Azzurra Orlandi, Anna Camporesi, Lucia Scarlato, Claudia Rossini, Roberto Russo, Antonello Sacco, Claudio Cafagno, Celeste Lidia Raguseo, Valentina Santoiemma, Anna Maria Musolino, Maria Chiara Supino, Anna Clemente, Luca Tagliaferri, Rosa Morello, Giandomenico Stellacci, Désirée Caselli, Danilo Buonsenso

**Affiliations:** 1 Department of Radiology, Giovanni XXIII Children Hospital, Azienda Ospedaliero Universitaria Consorziale Policlinico, 70126 Bari, Italy; 2 Pediatric Infectious Diseases Unit, Giovanni XXIII Children Hospital, Azienda Ospedaliero Universitaria Consorziale Policlinico, 70126 Bari, Italy; 3 Pediatric Anesthesia and Intensive Care, Vittore Buzzi Children’s Hospital, 20154 Milano, Italy; 4 Hospital University Pediatrics Clinical Area, Bambin Gesù Children’s Hospital IRCCS, 00165 Rome, Italy; 5 UOC Radioterapia Oncologica, Dipartimento Di Diagnostica Per Immagini, Radioterapia Oncologica Ed Ematologia, Fondazione Policlinico Universitario A. Gemelli, IRCCS, 00168 Rome, Italy; 6 Area Pediatrica, Dipartimento di Scienze Della Vita e Sanità Pubblica, Università Cattolica del Sacro Cuore, 00168 Rome, Italy; 7 Department of Woman and Child Health and Public Health, Fondazione Policlinico Universitario A. Gemelli IRCCS, 00168 Rome, Italy

**Keywords:** lung, ultrasound, pneumonia, children, *Mycoplasma pneumoniae*

## Abstract

**Aims:** To describe lung ultrasound (LUS) features of *Mycoplasma pneumoniae* pneumonia and their distribution in pediatric age, and to correlate imaging findings with clinical and laboratory data. **Methods:** This is a multicenter, prospective, pilot study that involved three hospitals. In total, 35 patients aged 1 month to 17 years, admitted with a diagnosis of *Mycoplasma pneumoniae* infection, were enrolled. History, clinical, microbiological, and ultrasound data were collected. The LUS examination was performed at admission, recording the following features: presence of subpleural consolidation, bronchograms, B lines, or pleural effusion, and their characteristics. The scans were performed using a standardized approach, in which a composite score was obtained by summing the scores of the different parameters. **Results:** Consolidations were seen in 97% of children (mostly located in basal, posterior, and lateral fields), and 65% of patients had multiple ones. Non-perilesional B lines were found in 43% of cases, principally in the posterior and basal fields. Pleural effusion was found in 37% of children. The univariate logistic regression showed a correlation between the age of the patient and large-sized consolidations. Moreover, increased lymphocyte count was associated with a lower risk of large-sized consolidations. **Conclusions:** LUS is a low-cost, non-invasive tool that can reveal findings suggestive of *Mycoplasma pneumoniae* infection and help physicians better manage children with lower respiratory tract infections, supporting a more personalized diagnostic and therapeutic approach, including antibiotic selection. These preliminary findings also indicate that a larger, comparative study involving other bacterial and viral etiologic agents is warranted to confirm whether LUS patterns are pathogen-specific and whether they can predict clinical outcomes.

## 1. Introduction

*Mycoplasma pneumoniae* is one of the most common causes of lower respiratory tract infections (LRTI) in children, accounting for 8% of community-acquired pneumonia (CAP) agents [1,2].

*Mycoplasma pneumoniae* infections occur most frequently during summer and early autumn [3], especially among children aged 5–17 years and young adults. 

*Mycoplasma pneumoniae* epidemic peaks tend to happen every 3–4 years. The Centers for Disease Control and Prevention (CDC) claimed that *Mycoplasma pneumoniae* infections have increased across the United States in 2024 from 1.0% to 7.2% among children aged 2–4 years, and from 3.6% to 7.4% among children aged 5–17 years [4]. Even in Europe, as early as autumn 2023, a peak in *Mycoplasma pneumoniae* infection had been recorded [5,6,7]. 

The severity of *Mycoplasma pneumoniae* infections ranges from mild to life-threatening. Macrolides are the first-line treatment for *Mycoplasma pneumoniae* infection, which is not susceptible to other antibiotics used to treat bacterial pneumonia, like penicillin. Hence, *Mycoplasma pneumoniae* early diagnosis would facilitate specific therapy.

The use of Lung Ultrasound (LUS) has become common in the diagnosis of CAP in pediatric age because it is low cost, radiation-free, and can be immediately available at the patient’s bedside, compared to Conventional X-Ray (CXR). Some studies tried to identify the most common LUS patterns in pediatric age for the etiological diagnosis of viral, bacterial, atypical, tubercular, and fungal pneumonia [8,9,10,11,12].

The most common LUS findings of atypical pneumonias in pediatric age are represented by small multiple bilateral subpleural consolidations, air bronchograms, especially superficial and dynamic, and vertical deep artifacts [11].

In 2012, the International Liaison Committee on Lung Ultrasound (ILC-LUS) published evidence-based recommendations for point-of-care lung ultrasound [13], reviewing the literature from 1966 to 2011. 

Many standardized protocols have been proposed for scoring lung ultrasound findings in various diseases, such as bronchopulmonary dysplasia [14], ARDS [15], or COVID-19 infection [16].

In 2020, Soldati et al. proposed an international standardized method of LUS for the management of patients with COVID-19. This method is based on the division of the patient’s chest into 14 areas (3 posterior, 2 lateral, and 2 anterior areas for each hemithorax) using reference points on the anatomical lines (paravertebral line, midaxillary line, internipple line, midclavicular line, spine line scapula, inferior angle of the line of the scapula). Furthermore, Soldati and collaborators identified a LUS score to quantify lung involvement, useful and reproducible in clinical practice for the diagnosis and monitoring of lung diseases [16].

The study aims to describe LUS findings of *Mycoplasma pneumoniae* LRTI and their distribution in the pediatric population, and to correlate them with clinical and laboratory findings. Pneumonia caused by *Mycoplasma pneumoniae* differs from typical bacterial pneumonia in both clinical presentation and treatment. Therefore, its prompt recognition is essential to ensure appropriate and effective therapy. Through the characterization of LUS features associated with *Mycoplasma pneumoniae* infection, this study supports the use of LUS as a helpful tool for early diagnosis.

## 2. Materials and Methods

### 2.1. Study Design

This study is part of an Italian multicenter, prospective, observational study called “Development of a Decision Support System Multiomic Software based on point-of-care clinical and lung ultrasound data for the personalized management of children with Community Acquired Pneumonia”, funded by the national resilience and recovery plan (PNRR). The study involves three Italian centers (Fondazione Policlinico Universitario A. Gemelli of Rome, Bambino Gesù Children’s Hospital of Rome, and Giovanni XXIII Children’s Hospital, Bari, Italy) and analyzes patients aged from 1 month to 17 years admitted between March 2023 and October 2024 with a clinical diagnosis of LRTI according to the British Thoracic Society guidelines [17]. The study was approved by the Ethics Committees of the centers. Informed consent was obtained from all participants and/or their legal guardians.

### 2.2. Inclusion and Exclusion Criteria

In total, 35 children, aged from 1 month to 17 years, were admitted between March 2023 and October 2024 with a suspected diagnosis of LRTI and confirmed *Mycoplasma pneumoniae* infection. Suspected diagnosis of LRTI required the presence of at least two of the following signs or symptoms: fever (>38 °C), cough, dyspnea, abnormal auscultatory findings, with or without abdominal pain. *Mycoplasma pneumoniae* infection was confirmed by either culture or molecular testing (polymerase chain reaction) on a pharyngeal swab or antibody serological test.

Exclusion criteria included patients with underlying diseases, such as respiratory tract anomalies (e.g., bronchopulmonary dysplasia or cystic fibrosis), immunodeficiency, cerebral palsy, neuromuscular diseases, congenital heart diseases, and malignancy. This choice was made in order to describe lung lesions related to *Mycoplasma pneumoniae*, without the confounding effects of other underlying conditions that could influence lung ultrasound findings.

For all 35 children included in this study, the history, clinical parameters, microbiological, and ultrasound data were collected. We recorded, respectively, the following data for each patient: clinical features (fever, cough, rhinitis, vomiting, diarrhea, dyspnea, abdominal pain, abnormal auscultatory findings), blood tests (complete and differential blood count, C-reactive protein, procalcitonin), chest X-ray (if performed), microbiological data as blood cultures and nasopharyngeal swabs to test culture, and multiplex polymerase chain reaction (PCR = panels for bacteria (i.e., *Streptococcus pneumoniae* and *Haemophilus influenzae*), viruses (i.e., respiratory syncytial virus, human rhinovirus, human bocavirus, influenza A and B viruses, parainfluenza viruses, adenovirus), and atypical bacteria (i.e., *Mycoplasma pneumoniae* and *Chlamydia pneumoniae*). In cases of doubt (clinical presentation suggestive of atypical pneumonia, with a negative nasopharyngeal swab), *Mycoplasma pneumoniae* IgM and IgG enzyme immunoassays (EIA) were performed.

The normal reference values considered for blood tests were the following:

‐ white blood cell count: normal if <15 × 10^9^ leukocytes/L;
‐ C-reactive protein: normal if <5 mg/L;
‐ procalcitonin: normal if <0.5 ng/mL.

Laboratory tests were performed using standard automated analyzers available at our institutions.

The microbiological diagnosis of acute LRTI by *Mycoplasma pneumoniae* was confirmed by the detection of *Mycoplasma pneumoniae* in nasopharyngeal swabs and/or antibody response to *Mycoplasma pneumoniae* (high IgM titers), if bacterial superinfection was excluded and clinical data were compatible with a diagnosis of atypical pneumonia.

### 2.3. Lung Ultrasound

Lung ultrasound (LUS) was performed within 12 h of the first clinical evaluation (T0) by a pediatrician or a pediatric radiologist. A linear probe (8–15 MHz) was used in most of the children; in some obese or older children, a convex probe (2.5–8 MHz) was used. The patients were positioned in the supine, lateral, and sitting positions.

The scans were performed according to the approach proposed by Soldati et al. in 2020 [16]. The scans need to be intercostal in the axial plane to cover the widest surface possible with a single scan. 

**Figure 1:**
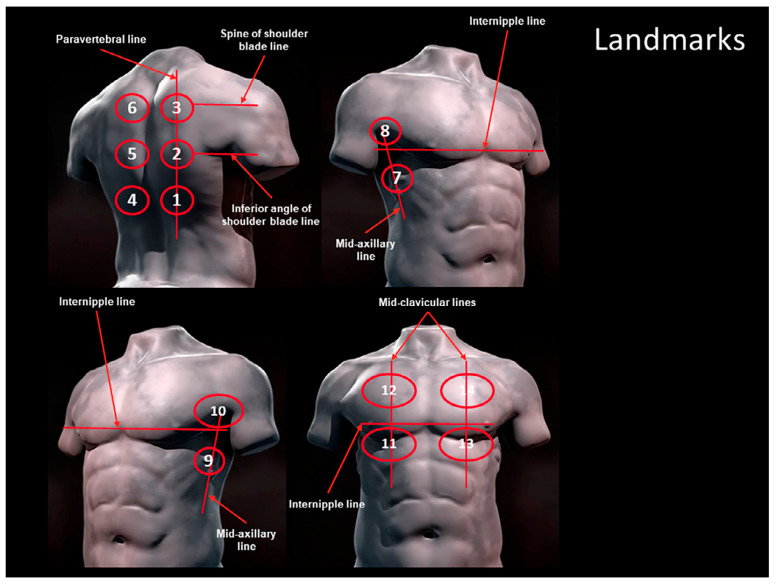
Image of schematic representation of the acquisition landmarks on chest anatomic lines. 1, 2, and 3 represent the right posterior areas; 4, 5, and 6 the left posterior areas; 7 and 8 the right lateral areas; 9 and 10 the left lateral areas; 11 and 12 the right anterior areas; and 13 and 14 the left anterior areas. Figure 1 is taken from the cited reference [16]. The copyright and license information in the article states: © 2020 by the American Institute of Ultrasound in Medicine. This article is being made freely available through PubMed Central as part of the COVID-19 public health emergency response. It can be used for unrestricted research re-use and analysis in any form or by any means with acknowledgement of the original source, for the duration of the public health emergency.

Thus, fourteen areas of the chest (Figure 1) were scanned.

To analyze the localization of LUS findings, we grouped lung fields as below:

‐ Right hemithorax lung fields group (1–3, 7–8, 11–12);
‐ Left hemithorax lung fields group (4–6, 9–10, 13–14);
‐ Posterior lung fields group (1–6);
‐ Lateral lung fields group (7–10);
‐ Anterior lung fields group (11–14);
‐ Basal lung fields group (1, 4, 7, 9, 11, 13);
‐ Middle-posterior lung fields group (2, 5);
‐ Upper lung fields group (3, 6, 8, 10, 12, 14).

The following LUS features were recorded: presence of subpleural lung parenchymal consolidation and its characteristics (single or multiple, unilateral or bilateral); presence of bronchograms, their characteristics (air or fluid) and position (superficial if close to the pleura or deep if ≥2 cm far from the pleura); presence of vertical artifacts or B lines and their characteristics (short or long, spared or confluents, unilateral or bilateral, perilesional or not); presence and type of pleural effusion as simple (anechogenic and dependent to gravity) or complex (presence of septa, hyperechogenic spot, not dependent to gravity) (Supplementary Material, Figures S1–S3).

A lung ultrasound score was obtained by summing scores of the following parameters (adapted from Soldati et al. [16]) (Table 1). The total LUS score can quantify the severity of pneumonia. A score of 0 is considered normal and corresponds to the presence of only A-lines or short vertical artifacts (no B-lines).

### 2.4. Statistical Analysis

Descriptive data are shown as absolute numbers and percentages for categorical variables, and median and interquartile range (IQR) for continuous ones. Categorical variables’ associations were studied with Chi-Squared Tests, while continuous variables’ associations were studied with the Kruskal–Wallis test. Logistic regression models were analyzed for selected outcomes, namely the presence of large consolidations (depth > 3 cm) and the presence of left lung non-perilesional B lines. We chose to use univariate logistic regression of the relatively small sample to avoid inaccurate estimates and overfitting. Data were analyzed with Stata 18.0 B.E. (StataCorp LLC, College Station, TX, USA). Two-tailed tests were used. *p*-values < 0.05 were considered significant.

## 3. Results

A total of 35 patients with confirmed *Mycoplasma pneumoniae* pneumonia were enrolled in this study: 17 females (48.6%) and 18 males (51.4%). The median age was 111 months (range 51–163).

Diagnosis of *Mycoplasma pneumoniae* infection was confirmed with positive nasopharyngeal swabs culture in 7 (20%) cases, positive nasopharyngeal swabs PCR in 25 (71%) cases, and high IgM titer in 3 (9%) cases. In two cases, nasopharyngeal swabs PCR was positive for *Mycoplasma pneumoniae* and Rhinovirus, and one case for *Mycoplasma pneumoniae*, Rhinovirus, and RSV.

The median value of the LUS score in our population of 35 children with *Mycoplasma pneumoniae* pneumonia was 17.

The main lung ultrasound findings at the time of diagnosis of *Mycoplasma pneumoniae* pneumonia were consolidation in 97% of children, non-perilesional B lines in 43%, and pleural effusion in 37%. The two major associations between ultrasound features were consolidations with non-perilesional B lines and consolidations with effusion (Table 2).

In total, 34 out of 35 patients (97%) had at least 1 lung field affected by consolidation, of which 22/34 (65%) had multiple consolidations, and 12/34 (35%) had a single one. According to the standardized LUS method proposed by Soldati et al. [16], most of the consolidations were located in the basal lung fields, both posterior and lateral ones. Fewer patients had consolidations in the middle-posterior, upper, and anterior lung fields (Table 3).

Furthermore, most of children (24/34, 71%) presented at least one right pulmonary field affected by consolidations, including the subgroup of 20/34 (59%) patients with only right unilateral consolidations; while 14/34 (41%) patients had at least one left pulmonary field affected by consolidations, including the subgroup of 10/34 (29%) with unilateral left consolidations. Only 4/34 (12%) patients presented bilateral consolidations (Table 3).

Analyzing the size of consolidations, we found a greater prevalence of medium-sized consolidations (1–3 cm) equal to 42%, followed by a lesser percentage of small consolidations (<1 cm) and large consolidations (>3 cm).

**Figure 2:**
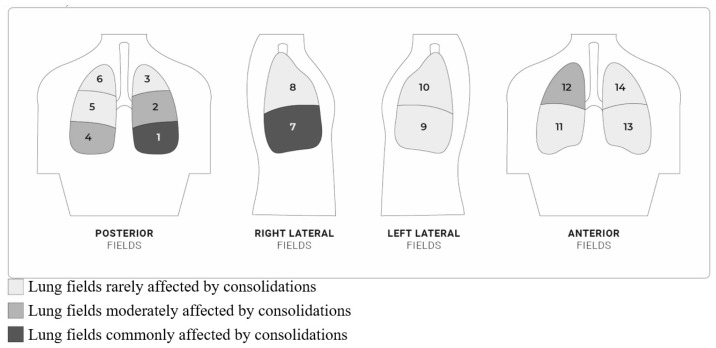
Description of location of consolidations in 14 lung fields (according to Soldati et al. method [16]) at admission. 1, 2, and 3 represent the right posterior areas; 4, 5, and 6 the left posterior areas; 7 and 8 the right lateral areas; 9 and 10 the left lateral areas; 11 and 12 the right anterior areas; and 13 and 14 the left anterior areas.

The analysis of consolidations in the 14 lung fields confirmed their prevalence in the right lung fields, the most affected being field 1 and field 7, then field 2, field 4, and field 12, then even less in all the remaining lung fields (see Figure 2).

Many patients presented with the association of consolidations and perilesional B lines (47%), while fewer patients presented with consolidations and perilesional white lung (37%).

The majority (65%) of children with consolidations had air bronchograms, which were superficial in 73% of cases and deep in 32%. Only 18% of children with consolidations presented fluid bronchograms.

Nearly half of the patients with consolidations (47%) had effusion, which in all cases was small and simple. Only two patients presented with effusion without consolidation.

Although less common than consolidations, non-perilesional B lines were also detected by LUS in 15 of 35 children (43%); most patients had unilateral non-perilesional B lines (Table 3). The most affected lung fields were the posterior and the basal ones. Minimal difference was detected between the presence of non-perilesional B lines in the left and right hemithorax (73% vs. 67%) (Table 3).

### Regression Models

Logistic regression models were applied to the following ultrasound outcomes: presence of any consolidation, presence of non-perilesional B lines in any of the lung fields, presence of small-medium or large size consolidations, presence of upper, middle-posterior, basal, anterior, lateral or posterior consolidations, presence of upper, middle-posterior, basal, anterior, lateral or posterior non-perilesional B lines, presence of effusion in any of the lung fields. Each ultrasound outcome was analyzed with age, symptoms such as fever, cough, rhinitis, conjunctivitis, dyspnea, vomiting, diarrhea, abdominal pain, blood laboratory values as WBC count (×10^9^ /L), neutrophils count (×10^9^ /L), lymphocytes count (×10^9^/L), monocytes count (×10^9^ /L), CRP (mg/L), procalcitonin (ng/mL).

The univariate logistic regression models analyzing clinical and laboratory factors for the presence of large consolidations in *Mycoplasma pneumoniae* pneumonia showed that children younger than 5 years had a protective factor for presenting large consolidations (OR 0.1; 95% CI: 0.011–0.908; *p* = 0.041). Moreover, increased lymphocyte count is associated with a lower risk of large size consolidations (OR 0.36; 95% CI: 0.13–0.96; *p* = 0.043) (see Table 4).

Furthermore, in the univariate logistic regression models, children younger than 5 years appeared to be associated with a greater risk of left lung non-perilesional B lines (OR 4.56; 95% CI: 0.975–21.322; *p* = 0.054) (see Table 4).

Logistic regression models analyzing other ultrasound outcomes did not show significant associations.

We conducted a comparative analysis between children younger than 5 years and those aged 5 years or older. All sonographic features identified by LUS were evaluated. Significant differences between the two groups were observed for large consolidations (greater than 3 cm), consolidations in the upper lung fields, and left-sided B-lines. Specifically, large consolidations were more frequently observed in children aged 5 years or older (*p* = 0.02), whereas consolidations in the upper fields and left-sided B-lines were more common in children younger than 5 years (*p* = 0.028 and *p* = 0.046, respectively) (Table 5). No significant differences were found for the remaining features, including the total LUS score.

## 4. Discussion

Since the 1990s, studies have been carried out in adult populations to find radiological and CT features of *Mycoplasma pneumoniae* pneumonia patterns [18]. From these studies, diffuse or localized interstitial involvement seemed to be the most common pattern, whereas lung consolidations appeared less suggestive of *Mycoplasma pneumoniae* infection [18]. 

In a prospective study in children hospitalized for *Mycoplasma pneumoniae* CAP, radiographic findings mainly included lung consolidations and single lobar infiltrates, while unilateral or bilateral multilobar infiltrates, pleural effusion, and hilar adenopathy were less frequent [2]. In a very recent study by Xiao et al. that compared low-dose CT scan with standard radiography, CT demonstrated a higher diagnostic rate, sensitivity, specificity, and diagnostic accuracy [19].

The importance of lung ultrasound (LUS) for the diagnosis and follow-up of LRTIs has been increasingly recognized, particularly because it allows accurate assessment without radiation exposure. Ciuca et al. recently proposed a newly developed LUS score for pediatric pneumonia, the PedPne score [20]. In our study, we applied a different LUS scoring system adapted from Soldati et al. [16], which was also used by Buonsenso and colleagues in a previous study on pediatric LRTIs [21]. Both scores consider consolidations, B-lines, and pleural effusion. The PedPne score evaluates 6 lung areas per hemithorax (12 total) [20], while the LUS score evaluates 7 areas per hemithorax (14 total) [16]. In PedPne, points are assigned based on a severity progressive scale from B-lines (1 point) to empyema (up to 9 points) [20]. In the LUS score, each lesion is evaluated in more detail: B-lines score from 1 point to 3 points, pleural effusion scores range from 1.5 to 3 points, and consolidations are scored from 4 to 10 points, based on their characteristics. Other authors, such as Stoicescu et al. [22] and Xie et al. [23], used other scores, to study the differences between bacterial (including *Mycoplasma pneumoniae*) and viral pneumonia, or to distinguish between *Mycoplasma pneumoniae* and viral pneumonias.

Buonsenso and others have also analyzed LUS features to discriminate bacterial, viral, and atypical LRTI in children, to support the etiological diagnosis and to avoid inappropriate antibiotic treatment [8,11,24].

Among the limitations of these previous studies, there is a lack of correlation between lung ultrasound findings and single pathogens of pneumonia groups.

In this study, we provided, to our knowledge, the most detailed and standardized description of LUS findings of *Mycoplasma pneumoniae* pneumonia, using a prospective and rigorous standardized protocol.

Our population of children hospitalized for *Mycoplasma pneumoniae* pneumonia presented a median age of 9 years, in line with data from other authors [2].

The median value of the LUS score in our population of children with *Mycoplasma pneumoniae* pneumonia was 17. We cannot compare this value with other studies because no one has previously used the Soldati method to perform LUS in *Mycoplasma pneumoniae* infection.

We showed that the main ultrasound finding at admission in children with *Mycoplasma pneumoniae* pneumonia was consolidations, followed by non-perilesional B lines, as also reported by Buonsenso et al. [11] and by Xie et al. [23].

Analyzing the data of consolidations, the size (in most cases included) was between 1 and 3 cm, confirming the results of Berce et al. [24], who supported that the size of consolidations in children with atypical pneumonia was approximately 20 mm, thus an intermediate between the significantly smaller consolidations of viral pneumonia (median diameter of 15 mm) and larger consolidations of bacterial pneumonia (median diameter of 30 mm).

According to Guo et al., the length and area of parenchymal lung consolidations are closely associated with the severity of *Mycoplasma pneumoniae* pneumonia in children [25]. 

Furthermore, we identified an interesting finding regarding pulmonary consolidations in our cohort: children younger than 5 years with *Mycoplasma pneumoniae* pneumonia appeared to have a lower risk of developing large consolidations. Jia et al. reported similar results, as in their study, the median age of children with pulmonary consolidations was significantly higher than that of children without consolidations [26]. A lower risk of large consolidations was also detected in children with increased lymphocyte count, in line with studies about neutrophil–lymphocyte ratio (NLR) [27,28,29] and with what Xie pointed out in a recent study [23].

We found that most of the consolidations were characterized by air bronchograms, which would make *Mycoplasma pneumoniae* pneumonia similar to bacterial pneumonia and different from viral pneumonia, as supported by Buonsenso et al. [11]. Furthermore, we found, above all, superficial air bronchograms, which appeared common in atypical pneumonia, while deep air bronchograms appear typical of viral pneumonia. Finally, among our patients, fluid bronchograms were very rare, as they are typical of bacterial pneumonia [11].

Our analysis showed that consolidations were mostly multiple and unilateral. Buonsenso et al. also found that most atypical pneumonias were unilateral, unlike viral pneumonias, which were more frequently bilateral, and bacterial pneumonias, almost exclusively unilateral [11]. 

In our study, consolidations were described, above all, in the right hemithorax, especially in the basal area, in both the posterior and lateral fields (Table 3, Figure 2). The consolidation size and distribution in *Mycoplasma pneumoniae* pneumonia can facilitate the etiological diagnosis of LRTI in pediatric age, comparing them with data obtained by LUS studies in bronchiolitis and viral and bacterial pneumonia [11,30,31,32,33,34,35]: in bronchiolitis consolidations are distributed mainly in the subpleural, posterior paravertebral and subscapular areas [30,31,33,36]; in viral pneumonias they appear to be bilateral, multifocal, especially in the posterior and lower lobes [22,37,38], while in bacterial pneumonias are unilateral, but the most affected area is debated [22,37,38].

In our population, the most common LUS finding after consolidations was non-perilesional B lines (43%). According to Buonsenso et al., diffuse deep vertical artifacts were significantly more common in atypical and viral pneumonia, compared to bacterial pneumonia [11]. In our study, non-perilesional B lines appeared commonly unilateral, and the most affected lung fields were the posterior and basal ones, with minimal differences between left and right hemithorax (Table 3). An inverse correlation between non-perilesional B lines and age was detected (Table 4). The increased risk of non-perilesional B-lines in younger children could be due to the distinct anatomical characteristics of the lungs at this age: immature immune defenses, a thinner epithelial barrier, and underdeveloped mucociliary clearance. These features, which are particularly pronounced in infants, predispose them to airway inflammation and obstruction [39].

Therefore, the differences observed between children above and below 5 years of age, both in regression models and in the comparison between the two groups, may be explained by age-related variations in the inflammatory response and the continuous maturation of the immune system throughout childhood. In older children, a more mature immune system may predispose them to a more pronounced inflammatory reaction [40,41] and consequently to the development of large focal consolidations, whereas the immune and anatomical characteristics of preschool children favor diffuse interstitial reactions, visible as B-lines.

In addition, the different risk of developing large consolidations between the two age groups may also be influenced by lung volume. Indeed, lung size varies with age, and not adjusting consolidation measurements for total lung volume represents a limitation of our study. Unfortunately, lung ultrasound does not allow a reliable estimation of lung volume, making such normalization unfeasible.

Furthermore, in our study, lung parenchyma around consolidations was characterized in half of the cases by perilesional B lines, and sometimes by perilesional white lung. Also, according to Buonsenso et al., perilesional B lines were more common in the atypical pneumonia group than in viral pneumonia [11].

In our cohort, nearly half of the patients with consolidations had effusion, which was small and simple in all cases. In the literature, pleural effusion has been rarely reported in atypical pneumonia (28%), but its presence appears to be a negative prognostic factor [12].

### Strengths and Limitations

Our prospective multicenter pilot study describes the LUS patterns observed in children with *Mycoplasma pneumoniae* pneumonia. Although these observations are consistent with previous descriptions of atypical pneumonia [11,24], they should be regarded as descriptive rather than disease-specific.

Unlike previous reports, our study employed the standardized 14-field LUS protocol proposed by Soldati et al. [16], providing a comprehensive and reproducible assessment of lung involvement. This approach enabled a detailed topographic evaluation and facilitated correlation with clinical and laboratory data, enhancing the consistency of findings across centers. Recognizing these ultrasound patterns may help clinicians consider *Mycoplasma pneumoniae* earlier in the diagnostic process and support a more judicious use of macrolides, while reducing unnecessary exposure to β-lactam antibiotics. Incorporating standardized LUS into clinical practice may also promote a radiation-free, patient-centered approach to pediatric CAP.

Our study has several limitations. The relatively small number of enrolled patients limited statistical power. Moreover, the absence of bacterial or viral control groups restricted our ability to compare LUS patterns across different pneumonia etiologies. However, in a previous study from our group involving a larger cohort of children with pneumonia of various origins, we compared LUS findings between bacterial and viral infections [21]. In that study, isolated or posterior confluent B-lines were identified as predictors of viral pneumonia, whereas large consolidations, pleural effusion, and fluid bronchograms were associated with typical bacterial pneumonia. Although these data derive from a broader cohort, they may provide a useful frame of reference when interpreting the descriptive findings observed in the present study on *Mycoplasma pneumoniae* pneumonia.

Despite adherence to a standardized protocol, some degree of inter-operator variability cannot be excluded. In addition, follow-up examinations were not systematically performed, limiting the assessment of the temporal evolution of LUS findings. These aspects highlight the need for a larger and well-characterized cohort, including bacterial and viral control groups, to validate and expand these preliminary observations and to establish standardized diagnostic criteria for atypical pneumonia. Longitudinal analyses on clinical outcomes are already ongoing within our cohort.

In this perspective, future research should involve children with pneumonia caused by different pathogens, applying the same standardized protocol used in this pilot study. This would allow comparison of the LUS features observed in *Mycoplasma pneumoniae* infection with those seen in pneumonia due to other bacteria (e.g., *Streptococcus pneumoniae, Haemophilus influenzae*) or viruses (e.g., Rhinovirus, RSV), and to understand whether these patterns are truly specific. If confirmed, these differences would allow LUS to support clinicians in choosing the most appropriate and personalized antibiotic therapy. Future research should also explore whether ultrasound findings could predict short- and long-term clinical outcomes, such as need for respiratory support, admission to intensive care, length of stay, or the development of complications, in order to better define the clinical and prognostic value of LUS.

## 5. Conclusions

We described the most frequent ultrasound findings in a cohort of patients with pneumonia caused by *Mycoplasma pneumoniae* infection: predominantly unilateral, multiple, medium-sized consolidations, most commonly located in the basal lung fields and accompanied by superficial air bronchograms. In approximately half of the cases, these lesions were also associated with a small, simple pleural effusion. To our knowledge, this is the first study to exclusively describe lesions caused by *Mycoplasma pneumoniae* and to examine these lesions in detail according to a precise protocol.

In conclusion, considering all the advantages of LUS and its high sensitivity and specificity in the study of LRTI in pediatric age, a new frontier can be represented by its use to support diagnosis in children with respiratory infections. Our study demonstrates that, by using a standardized method and scoring system, it is possible to identify LUS findings suggestive of *Mycoplasma pneumoniae* infection. The integration of these imaging data with clinical and laboratory findings can provide valuable support for the etiological diagnosis of pneumonia. Further case–control studies will be necessary to confirm and expand our findings.

## Figures and Tables

**Table 1:** LUS score adapted from Soldati et al. [16].

Parameters		Points					
		0	0.5	1	2	3	4
**B lines**				Isolated	Confluent	White lung	
**Consolidation**	Presence	Absent					Present
	Size (depth)	<1 cm		1–3 cm	>3 cm		
	Location			Unilateral	Bilateral		
**Air bronchograms**				Superficial (<2 cm from pleural line)	Deep (>2 cm from pleural line)		
**Effusion**	Size		Small (<1 cm)	Moderate/large (>1 cm)			
	Type			Simple	Complex		

**Table 2:** Main findings of LUS performed at admission of children with *Mycoplasma pneumoniae* pneumonia in at least one lung field and associations of LUS findings.

LUS Findings in at Least One Lung Field	Total = N. 35 (%)	*p*-Value
Consolidation	34 (97%)	
Non-perilesional B lines	15 (43%)	
Pleural effusion	13 (37%)	
***Coexisting Lung Ultrasound Findings***		
Non-perilesional B lines and consolidation	14 (40)	*p* = 0.833
Non-perilesional B lines and effusion	4 (11)	*p* = 0.267
Isolated B lines and confluent B lines	1 (3)	*p* = 0.077
Isolated B lines and consolidation	2 (6)	*p* = 0.720
Isolated B lines and effusion	1 (3)	*p* = 0.698
Confluent B lines and consolidation	4 (11)	*p* = 0.601
Confluent B lines and effusion	1 (3)	*p* =0.593
Consolidation and effusion	13 (37)	*p* = 0.263

**Table 3:** Description of children with at least one lung field (according to the Soldati et al. approach [16]) affected by consolidation or non-perilesional B Lines at admission for *Mycoplasma pneumoniae* pneumonia.

Lung Field Groups	Consolidation	Non-Perilesional B-lines
Posterior (1–6)	25 (73.5%)	11 (73%)
Lateral (7–10)	19 (55.8%)	8 (53%)
Anterior (11–14)	12 (35.2%)	5 (33%)
Basal (1, 4, 7, 9, 11, 13)	27 (79.4%)	10 (67%)
Middle-posterior (2, 5)	13 (38.2%)	5 (33%)
Upper (3, 6, 8, 10, 12, 14)	13 (38.2%)	8 (53%)
Left hemithorax lung fields group (4–6, 9–10, 13–14)	14 (41)	11 (73%)
Right hemithorax lung fields group (1–3, 7–8, 11–12)	24 (71)	10 (67%)
Left and right hemithorax lung field groups (4–6, 9–10, 13–14; 1–3, 7–8, 11–12)	4 (12%)	6 (40%)
Total	34	15

**Table 4:** Results of the univariate logistic regression models analyzing clinical and laboratory factors for large-size consolidations and left lung non-perilesional B lines in *Mycoplasma pneumoniae* pneumonia.

	Large Size Consolidation			Left Lung Non-Perilesional B Lines		
	OR	95% CI	*p*	OR	95% CI	*p*
**Age** (<5 vs. ≥5 years old)	0.1	0.011–0.908	0.041	4.56	0.975–21.322	0.054
**Fever** (yes vs. no)	2.66	0.26–26.86	0.405	2	0.197–20.33	0.558
**Cough** (yes vs. no)	0.57	0.03–9.98	0.701	1		
**Rhinitis** (yes vs. no)	1			2.62	0.436–15.81	0.292
**Conjunctivitis** (yes vs. no)	1			1		
**Dyspnea** (yes vs. no)	1			0.844	0.137–5.220	0.856
**Vomiting** (yes vs. no)	0.833	0.068–10.202	0.887	1		
**Diarrhea** (yes vs. no)	1			1		
**Abdominal pain** (yes vs. no)	0.833	0.068–10.202	0.887	1.1	0.089–13.592	0.941
**WBC count** (for 1 × 10^9^/L more)	0.878	0.759–1.017	0.082	0.973	0.870–1.090	0.639
**Neutrophil count** (for 1 × 10^9^/L more)	0.913	0.788–1.059	0.230	0.930	0.796–1.087	0.363
**Lymphocyte count** (for 1 × 10^9^/L more)	0.36	0.13–0.96	0.043	1.054	0.653–1.700	0.831
**Monocyte count** (for 1 × 10^9^/L more)	0.251	0.024–2.680	0.253	0.691	0.136–3.508	0.656
**CRP** (for 1 mg/L more)	1.007	0.992–1.022	0.342	1.007	0.992–1.022	0.372
**Procalcitonin** (for 1 ng/mL more)	1.268	0.670–2.400	0.466	0.858	0.4731.554	0.613

**Table 5:** Comparison between patients ≥5 years old and patients <5 years old.

		≥5 Years Old	<5 Years Old	*p*-Value
		N = 24	N = 11	
**Large consolidations**	No	12 (50.0%)	10 (90.9%)	0.02
	Yes	12 (50.0%)	1 (9.1%)	
**Consolidations in upper fields**	No	18 (75.0%)	4 (36.4%)	0.028
	Yes	6 (25.0%)	7 (63.6%)	
**Left B-lines**	No	19 (79.2%)	5 (45.5%)	0.046
	Yes	5 (20.8%)	6 (54.5%)	

## Data Availability

The datasets generated during and/or analyzed during the current study are available from the corresponding author upon reasonable request.

## Supplementary Materials

The following supporting information can be downloaded at: https://www.mdpi.com/article/10.3390/children12121669/s1, Figure S1: Medium-sized (1–3 cm) lung subpleural consolidation with superficial air and fluid bronchograms (a), and medium-sized lung parenchymal consolidation with moderate-large (>1 cm) simple pleural effusion (b); Figure S2: Non-perilesional confluent B Lines (a), perilesional areas of white lung with small subpleural consolidations (b), and non-perilesional white lung with pleural effusion (c); Figure S3: Small-sized (<1 cm) simple pleural effusion associated with parenchymal consolidation.
