# Supplementary material for: Lung Ultrasound Findings in Pediatric Mycoplasma Pneumoniae Pneumonia: A Prospective Multicenter Pilot Study
Source: Children (Basel). 2025 Dec 8;12(12):1669. doi: 10.3390/children12121669 (PMC12731742; doi:10.3390/children12121669)
Supplement: Supplementary file 1 [file children-12-01669-s001.zip › children-4003869-supplementary.pdf]

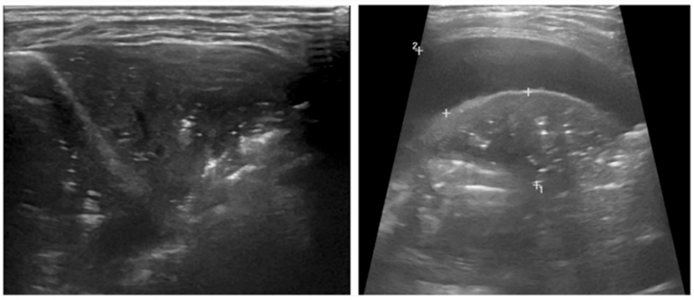

a b

Figure S1. Medium-sized (1-3 cm) lung subpleural consolidation with superficial air and fluid bronchograms (a), and medium-sized lung parenchymal consolidation with moderate-large (>1 cm) simple pleural effusion (b).

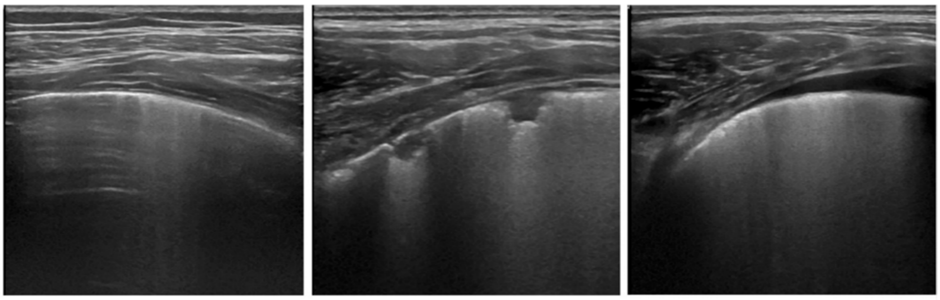

a b c

Figure S2. Non-perilesional confluent B Lines (a), perilesional areas of white lung with small subpleural consolidations (b) and non-perilesional white lung with pleural effusion (c).

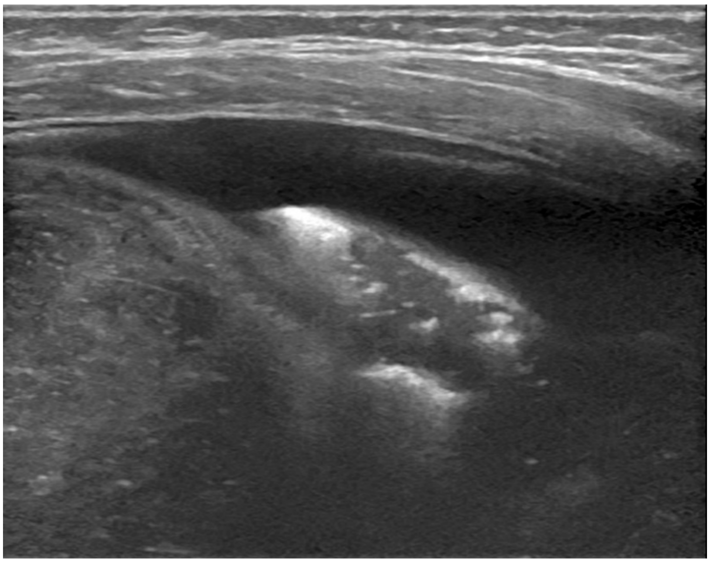

Figure S3. Small-sized (<1 cm) simple pleural effusion associated with parenchymal consolidation.
